# Deep invasive mucinous adenocarcinoma without surface amorphous pattern on inflammatory bowel disease: specific disease with specific characterization

**DOI:** 10.1055/a-2363-0905

**Published:** 2024-07-29

**Authors:** Elena De Cristofaro, Clara Yzet, Louis-Jean Masgnaux, Jean Grimaldi, Jérôme Rivory, Tanguy Fenouil, Mathieu Pioche

**Affiliations:** 1 9318 Gastroenterology Unit, Department of Systems Medicine, University of Rome Tor Vergata, Roma, Italy; 2 Department of Gastroenterology, Amiens University Hospital, Amiens, France; 3 Gastroenterology and Endoscopy Unit, Edouard Herriot Hospital, Hospices Civils de Lyon, Lyon, France; 4 Institute of Pathology, Groupement Hospitalier Est, Hospices Civils de Lyon, Lyon, France

Endoscopic characterization of deep invasive carcinoma using current classifications (CONECCT III, JNET III, NICE III) is effective but was not developed for neoplasias associated with inflammatory bowel diseases (IBDs). In IBD, dysplastic lesions often present as flat, non-granular laterally spreading tumors (LST-NGs) 1 2 . However, mucosal distortion caused by chronic inflammation and regenerative changes can conceal dysplasia, making the detection and characterization of the colonic lesions challenging 3 4 . The present case illustrates that deep invasive adenocarcinoma can occur with very slight mucosal changes in an IBD lesion ( Video 1 ).

Invasive adenocarcinoma in inflammatory bowel disease lesion with slight mucosal changes.Video 1

A 53-year-old woman was referred for endoscopic resection of a 15-mm IBD-related sigmoid lesion (previously diagnosed with high grade dysplasia on biopsy). The lesion was classified as LST-NG with a single and delineated area exhibiting a disorganized pattern ( Fig. 1 ). Endoscopic submucosal dissection (ESD) was indicated to ensure en bloc resection, and an adaptative traction strategy with A-TRACT was employed due to an expected strong fibrosis. The dissection phase proceeded smoothly until reaching the center of the lesion, where a pool of mucus emerged and spread out within the submucosal region. The procedure ended without adverse events facilitated by the use of an adaptative traction device and an underwater strategy during the dissection. The histopathology revealed an adenocarcinoma with submucosal invasion (R1 vertical margins and high risk features) and a mucinous lake within the lesion ( Fig. 2 ).

**Fig. 1:**
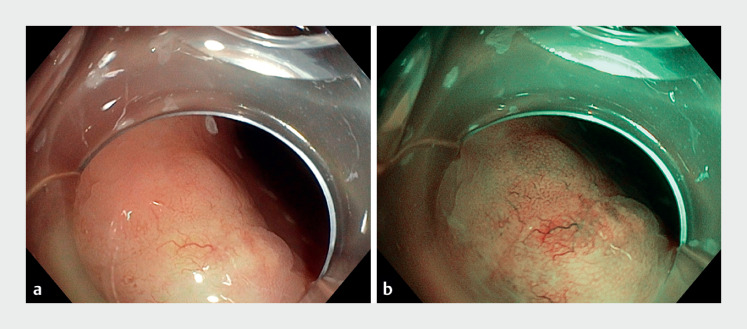
Non-granular laterally spreading tumor with a single and well-defined area exhibiting a disorganized pattern. **a** White light endoscopy. **b** Narrow band imaging.

**Fig. 2:**
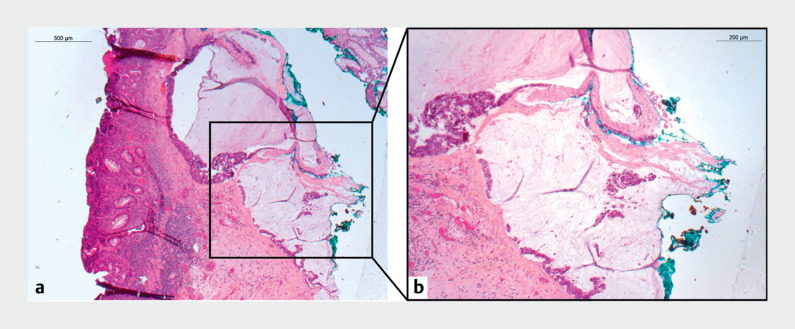
**a** Histopathological examination at low power of the endoscopic submucosal dissection specimen found mucosal inflammatory changes admixed with few adenocarcinoma glands invading the lamina propria. They were associated with deep submucosal infiltrating glands floating in mucin pools. **b** At higher magnification, the neoplastic glands presented typical characteristics of invasive mucinous adenocarcinoma and reached the endoscopic resection margin.

It can be inferred that the whitish cloudy appearance in the submucosa may signal the presence of a mucinous component, implying a deeply invasive lesion with high risk features such as mucinous submucosal invasion. Consequently, it is frequently linked with a non-curative resection and could lead to stopping the dissection during the procedure. The challenge in characterizing IBD lesions should prompt the utilization of progressively advanced endoscopic technologies and techniques to ensure en bloc resection with an accurate histological evaluation.

Endoscopy_UCTN_Code_TTT_1AO_2AG_3AD

## Notes

Endoscopy E-Videos https://eref.thieme.de/e-videos
*E-Videos* is an open access online section of the journal *Endoscopy* , reporting on interesting cases and new techniques in gastroenterological endoscopy. All papers include a high-quality video and are published with a Creative Commons CC-BY license. Endoscopy E-Videos qualify for HINARI discounts and waivers and eligibility is automatically checked during the submission process. We grant 100% waivers to articles whose corresponding authors are based in Group A countries and 50% waivers to those who are based in Group B countries as classified by Research4Life (see: https://www.research4life.org/access/eligibility/ ). This section has its own submission website at https://mc.manuscriptcentral.com/e-videos .
